# Identification of Endemic Region for Severe Fever with Thrombocytopenia Syndrome in an Alluvial Plain of Hebei Province, China

**DOI:** 10.3390/v17060854

**Published:** 2025-06-16

**Authors:** Yanan Cai, Yamei Wei, Luling Li, Minghao Geng, Yan Zheng, Xinyang Zhang, Zhanying Han, Yanbo Zhang, Yonggang Xu, Xu Han, Qi Li

**Affiliations:** 1 Institute for Viral Disease Prevention and Control, Hebei Provincial Center for Disease Prevention and Control, Shijiazhuang 050021, China; yanan589@163.com (Y.C.); weiyamei2013@163.com (Y.W.); gmhcdc@126.com (M.G.); zxhuade@126.com (X.Z.); hzhyehf@163.com (Z.H.); zhyb1966@126.com (Y.Z.); walterxu04@sina.com (Y.X.); 2 School of Public Health, Hebei Medical University, Shijiazhuang 050017, China; tl596223969@163.com; 3 Hebei Key Laboratory of Pathogens and Epidemiology of Infectious Diseases, Hebei Provincial Center for Disease Prevention and Control, Shijiazhuang 050021, China; 4 Cangzhou Center for Disease Prevention and Control, Cangzhou 061014, China; bmcgjc2010@126.com

**Keywords:** endemic region, SFTS, epidemiological characteristics, SFTSV antibodies, molecular characterization, Hebei

## Abstract

Severe fever with thrombocytopenia syndrome (SFTS), an emerging infectious tick-borne viral disease, is increasingly affecting human beings worldwide. SFTS monitoring has been carried out since 2010 in mainland China. Since 2022, an increase in local cases has been noted in the central coastal plain region of Hebei Province. This study aimed to identify the endemic region in the central coastal plain region by epidemiological characteristics, antibody surveillance and molecular characterization. Case data were obtained from the Chinese Disease Control and Prevention Information System. Serum samples from suspected or clinically diagnosed cases, the indigenous healthy population and native domesticated animals were collected for laboratory tests, along with ticks in the central coastal plain region of Hebei Province, China. The local cases were mainly distributed in Cangzhou City, located at the central coastal plain region of Hebei Province. The 0.68% of IgM antibody detection rate and 1.71% of IgG antibody detection rate in this study showed the potential existence of subclinical or mild infections in Cangzhou. Phylogenetic analysis indicated that all sequences from patients, ticks and sheep clustered within the F subtype, exhibiting a close evolutionary relationship and the possible circulation of SFTSV having established among animal hosts and ticks in Cangzhou. These findings first identify the natural focus of SFTSV in the central plain region of Hebei Province, highlighting enhanced surveillance measures for preventing and controlling SFTSV.

## 1. Introduction

Severe fever with thrombocytopenia syndrome is a recently identified tick-borne infectious disease with a high case mortality risk, ranging from 6 to 40% [1,2,3]. Its primary clinical manifestations include an emerging hemorrhagic fever, thrombocytopenia, leukopenia, central nervous system symptoms and gastrointestinal symptoms [4,5]. SFTS was first reported in 2009 in rural areas of Hubei Province and Henan provinces, with severe fever with thrombocytopenia syndrome virus (SFTSV) as the pathogen [6,7]. SFTSV is a single-stranded negative-chain RNA virus characterized by a tripartite genome (L, M and S segments). SFTSV is also referred to as Dabie bandavirus (DBV), recently reclassified into the class *Bunyaviricetes*, order *Hantavirales*, family *Phenuiviridae* and genus *Bandavirus* by the International Committee on Taxonomy of Viruses (ICTV, https://talk.ictvonline.org) in the 2023 taxonomic update.

As of 2021, SFTS cases have been identified in at least 27 provinces in China, with 12,953 confirmed cases, according to surveillance data from the Chinese Center for Disease Control and Prevention (CDC) [8]. Most cases are mainly prevalent in seven central-eastern provinces, with the endemic areas expanding from central regions to the northeast, west and south [9]. In China, confirmed cases of SFTS are defined by the presence of at least one of the following criteria: positive nucleic acid detection of SFTSV in case specimens; a fourfold or greater increase in SFTSV IgG antibody titers between convalescent and acute-phase sera; or isolation of SFTSV from case specimens. Furthermore, confirmed SFTS cases have been documented in other Asian countries, including Republic of Korea [10], Japan [11], Vietnam [12],Pakistan [13], Myanmar [14] and Thailand [15]. As of now, there is no licensed vaccine or therapeutic treatments against SFTSV. In 2017, SFTS was listed as one of the ten most critical infectious diseases because of a serious threat to public health and safety [16].

Tick bites are thought to be the primary transmission route for SFTSV infection, and *Haemaphysalis longicornis* serves as the principal vector [4,17]. SFTSV can also be transmitted through exposure to blood or other bodily fluids from infected patients [18,19,20]. In addition, some wild animals and domestic animals, such as rodents, cattle sheep, dogs and pigs can act as amplifying hosts for SFTSV [21,22,23]. The natural focus is the basis for the survival, persistence and transmission of SFTSV, constituting a potential epidemiological threat [24]. Therefore, the early identification of their existence and localization is critical for implementing preventive or control measures.

The natural focus of SFTS was generally considered to exist in hilly and mountainous areas. However, clustered SFTS cases have been reported in some plain areas [24]. In recent years, patients with SFTS have been increasingly found in the central alluvial plain region of Hebei Province. However, the transmission of SFTSV infection in Hebei Province has not been well investigated until now. Furthermore, the characterization of molecular features of SFTSV in Hebei remains elusive.

In this study, we revealed the existence of Hebei Province as an SFTSV endemic region, and we used epidemiological investigation and laboratory testing from multiple perspectives, including epidemiological characteristics, antibody surveillance and molecular characterization, to further verify that SFTSV natural focus existed in the alluvial plain areas.

## 2. Materials and Methods

### 2.1. Ethics Statement

This investigation was conducted to collect epidemiological data on SFTS from 2015 to 2024. Patients’ serum samples were obtained between 2022 and 2024, while serum samples from healthy individuals, along with tick and domestic animal samples, were collected in 2024. All samples were collected before this study during public health investigations of SFTS and were examined as anonymous samples. The study protocol was approved by the Medical Ethics Committee of Hebei Medical University (No. 2024012) and adhered to Chinese medical research regulations.

### 2.2. Study Area

**Figure 1:**
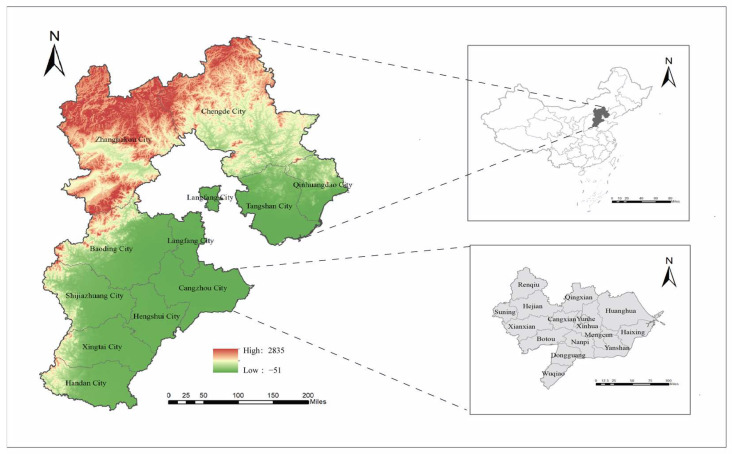
Map of Hebei Province in China. Map source: The Chinese Academy of Surveying and Mapping.

Hebei (36°05′ N–42°40′ N, 113°27′ E–119°50′ E) is a northern plain-dominated province of China and comprises 11 cities and 168 counties. Hebei Province is predominantly characterized by plains, with mountainous and plateau terrain in the northwest, including the Taihang and Yanshan Mountains, while the southeast consists of low-lying alluvial plains. Cangzhou City is located in southeastern Hebei Province, characterized by a coastal alluvial–lacustrine plain (Figure 1).

### 2.3. Data Collection

Data on SFTS cases were collected from the infectious disease surveillance module of the China Information System for Disease Control and Prevention (CISDCP). The gathered information included age, sex, occupation, case type, date of diagnosis, reporting area and living address. The population data for each year were obtained from the basic information module of the CISDCP.

### 2.4. Sample Collection

#### 2.4.1. Clinical Sample Collection

This study enrolled patients who were suspected of having or were clinically diagnosed with SFTS in Cangzhou from January 2022 to December 2024 in accordance with the diagnostic criteria for SFTS established by the Chinese Ministry of Health. Three to five milliliters of blood samples was collected at the acute phase of suspected SFTS cases by the local CDC or hospital.

Blood samples without anticoagulant were collected into micro-centrifuge tubes and allowed to stand and clot at room temperature for 3 h. After clotting, the samples were centrifuged at 3000× *g* for 10 min at 4 °C. The serum was then carefully separated and stored at −80 °C immediately until further use.

#### 2.4.2. Healthy Individuals’ Serum Sample Collection

Serological surveillance was conducted in Yanshan County and Haixing County of Cangzhou City, where SFTS cases had been reported. Healthy individuals were divided into four age groups as 0–19, 20–39, 40–59 and ≥60. The number of samples collected in each village was not less than 100. The amount of non-anticoagulated serum specimens should not be less than 5 mL, separated in time, divided into 2 tubes, and stored at −80 °C. One tube was used for laboratory serological antibody testing, while the other was reserved for verification purposes.

#### 2.4.3. Tick and Domestic Animal Sample Collection

Ticks were directly collected from infested animals (sheep, dogs and cats) or collected by flagging from vegetation between June to October 2024 in Yanshan County of Cangzhou City. All collected ticks were maintained alive at room temperature before being frozen for specimen preservation. Tick species were identified morphologically [25].The collected ticks were divided into 32 pools according to tick species and host sources, with 6 to 15 ticks in each pool. The ticks in each pool were washed three times with phosphate-buffered saline (PBS). One milliliter of Dulbecco’s modified Eagle’s medium (DMEM) and grinding beads were added to the ticks in each pool, and the ticks were ground by a high-throughput tissue homogenizer at a low temperature to completely homogenize them.

Blood samples of domestic animals were collected from those raised by patients with SFTS in Cangzhou city. Approximately 1–2 mL of blood samples was collected from jugular veins depending on body size, and serum was obtained after blood clotting, then stored in boxes with ice packs and transported to the laboratory along with the ticks. All ticks and blood samples were stored at −80 °C until further processing.

### 2.5. Detection of SFTSV by Quantitative Real-Time PCR

Viral RNA was extracted from serum samples of patients and domestic animals with SFTS, and tick suspensions, using a nucleic acid isolation kit (Jiangsu Bioperfectus Technologies Co., Ltd., Taizhou, China), following the manufacturer’s instructions. The real-time RT-PCR assay was performed using the PCR Diagnostic Kit for SFTSV RNA (GuangZhou Daan Gene Technology Co., Ltd., Guangzhou, China) according to the manufacturer’s instructions. Data were analyzed using the software supplied by the manufacturer.

### 2.6. SFTSV Antibody Detection

Serum samples from healthy individuals were tested for SFTSV-specific IgM and IgG antibodies using an ELISA kit (Zhongshan Bio-Tech Co., Ltd., Zhongshan, China) based on the procedures previously described [26,27]. In brief, 100 μL of diluted serum, 100 μL of horseradish peroxidase (HRP)-labeled enzyme conjugate and the chromo-genic substrate were added to each well. Finally, the optical density (OD) value was read at 450 nm with an Epoch microplate reader (BioTek Inc., Winooski, VT, USA) with Gen5 software (Version 1.10, BioTek). The cut-off value was set at the average OD value of the negative controls plus 0.10.

The microneutralization assay was applied to detect neutralizing antibodies in SFTSV IgG seropositive samples from healthy individuals. The serum samples were serially diluted twofold with DMEM diluent, mixed with an equal volume of SFTS virus at 25 TCID_50_/50 μL and incubated at 4 °C overnight for neutralization. The mixture of virus and serum was then added to VERO cells and cultured in a 37 °C CO_2_ incubator for 7 days. The cells were fixed with 80% acetone for 20 min, followed by the addition of an enzyme-labeled antibody against SFTS virus and incubation at 37 °C for 45 min. Finally, the absorbance value was measured at 450 nm. The neutralizing antibody titer of the serum is the reciprocal of the highest dilution of the serum that exhibits a positive neutralization reaction.

### 2.7. Whole Genome Sequencing

Positive samples (Ct ≤ 28) underwent whole genome amplification using the RT-PCR with specific primers previously described [28,29]. First-strand cDNA was amplified using SuperScript™ IV First-Strand Synthesis System (Thermo Fisher Scientific, Waltham, MA, USA), according to the manufacturer’s instructions. First-strand cDNA synthesis was performed under the following cycling conditions: 25 °C (10 min), 50 °C (10 min) and 85 °C (5 min). Second PCR amplification used 3 µL of the first-round product, 12.5 µL GoTaq^®^ Colorless Master Mix, 7.5 µL nuclease-free water and 2 µL primer, with the following cycling parameters: 95 °C (5 min), 35 cycles of 94 °C (30 s), 52 °C (45 s), 72 °C (1 min) and a final extension at 72 °C (10 min). Sequencing was conducted on a NextSeq 2000 platform (Illumina, San Diego, CA, USA).

### 2.8. Phylogenetic Analysis

Sequences of SFTSV strains were aligned with GenBank references using Clustal W in MEGA 11. Maximum likelihood trees were constructed using the best-fit substitution model, and branch support was calculated based on 1000 bootstrap replicates. Reference sequences were selected based on genomic completeness (full-length L, M, S segments), genotype (A, B, C, D, E, F) and geographic relevance (China, Japan, Republic of Korea).

### 2.9. Statistical Analysis

Chi-squared tests were conducted to analyze differences in socio-demographic characteristics of patients with SFTS and antibody detection rates in healthy individuals’ serum samples, with significance defined as *p* < 0.05.

## 3. Results

### 3.1. Overview of SFTS in Hebei Province

**Figure 2:**
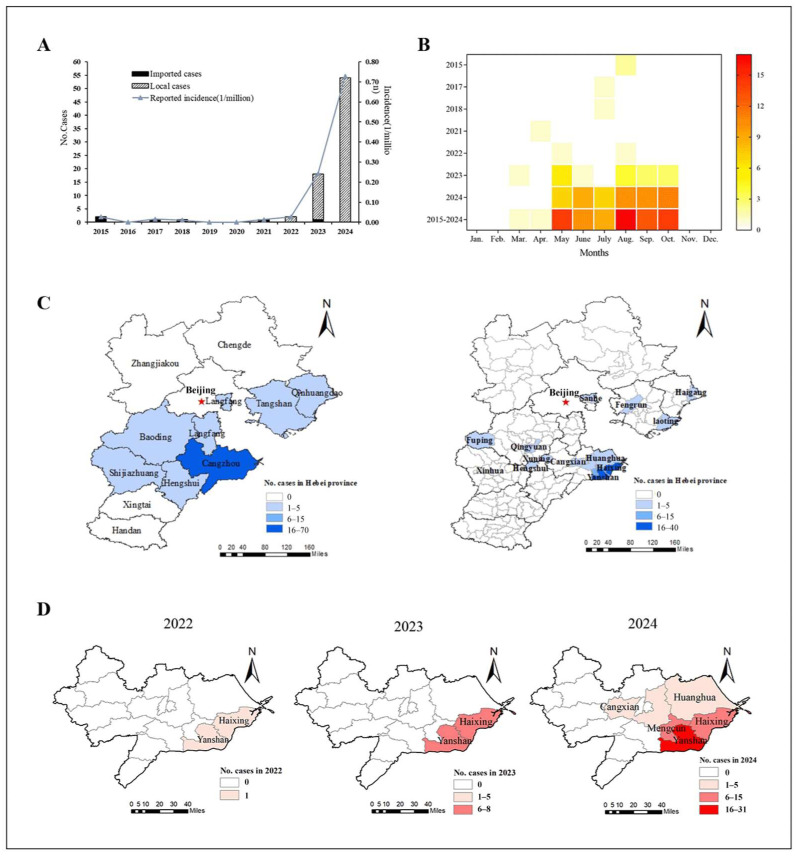
Epidemiological characteristics of SFTS cases in Hebei Province. (**A**) Temporal distribution of imported and local SFTS cases. (**B**) Monthly reported distribution of SFTS cases. (**C**) **Left**: Geographical distribution of SFTS cases at city level from 2015 to 2024. **Right**: Geographical distribution of SFTS cases at county level from 2015 to 2024. (**D**) Geographical distribution of SFTS cases in Cangzhou from 2022 to 2024.

From 2015 to 2024, a total of 79 SFTS cases involving 70 lab-confirmed cases and 9 clinically diagnosed cases were reported in six cities of Hebei Province, with four deaths. The average annual fatality rate was 5.06% across the entire province. The number of SFTS cases showed an increasing trend between 2015 (2 cases) and 2024 (54 cases). The first SFTS case in Hebei Province was reported in 2015. From 2015 to 2021, all SFTS cases were imported. Local cases were first reported in 2022, and then the number of reported cases has increased significantly (Figure 2A).

### 3.2. Epidemiological Characteristics of SFTS Cases

#### 3.2.1. Temporal Distribution

According to the overall incidence from 2015 to 2024, the SFTS cases occurred between March and October, but were mainly distributed between May and October (97.5%, 77/79), peaking in August (Figure 2B). The cases reported from August to October accounted for 55.7% (44/79), showing significant seasonal characteristics.

#### 3.2.2. Regional Distribution

From 2015 to 2024, 79 cases of SFTS (including both local and imported cases) were reported in Cangzhou (70 cases), Baoding (3 cases), Tangshan (2 cases), Langfang (1 case), Shijiazhuang (1 case), Qinhuangdao (1 case) and Hengshui (1 case). The imported cases were distributed in Baoding, Tangshan, Hengshui and Langfang, with one case each. The local cases were mainly distributed in Cangzhou City, which accounted for 92.0% (69/75), and Baoding, Shijiazhuang, Tangshan and Qinhuangdao City, with one case each (Figure 2C). At the town level, the number of locally reported regions endemic for SFTS in Cangzhou increased from two (2022) to five towns (2024), showing significant regional clusters (Figure 2D). Especially, the largest number of cases were distributed in Yanshan and Haixing Counties of Cangzhou City, with 40 and 20 cases reported, respectively.

#### 3.2.3. SFTS Population Distribution

**Figure 3:**
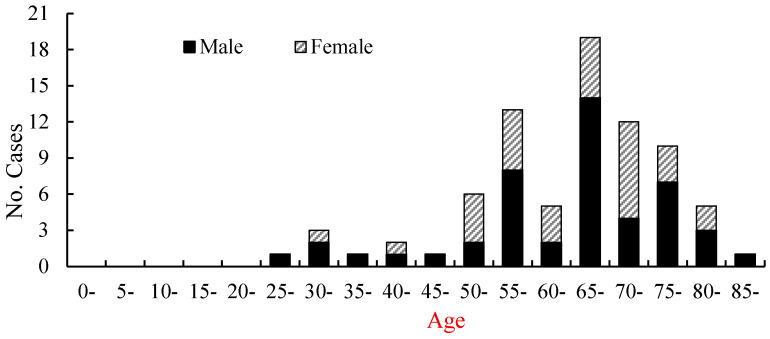
The age and gender distribution of SFTS cases in Hebei Province from 2015 to 2024.

During the observed period, the youngest case was a 27-year-old male and the oldest case was an 87-year-old male, with an average age of 64 years. The majority of cases occurred in middle-aged and elderly individuals. Among them, the 50–84-year-old age group accounted for 88.6% (70/79) of the total SFTS cases (Figure 3). Among the SFTS cases, there were 47 males and 32 females, with an overall male-to-female ratio of 1.47:1. The SFTS occupational distribution showed that 87.3% (69/79) were farmers, followed by the unemployed (6.4%).

### 3.3. SFTSV Detection in Patients, Ticks and Animals

From 2022 to 2024, a total of 81 suspected and clinically diagnosed cases were collected, including 4 cases that were reported from other regions. SFTSV RNA detection revealed that 50 cases were positive for SFTSV, with a positivity rate of 61.73%. Among these, 33 specimens with Ct values ≤ 28 were eligible for whole-genome sequencing.

In 2024, a total of 403 ticks (101 ticks from vegetation and 302 ticks from hosts) and 73 domestic animals (31 pigs, 20 cattle and 22 sheep) were collected in villages where SFTS cases were reported. Six to fifteen ticks from different host animals or ticks from vegetation were respectively pooled and tested for SFTSV RNA. Two ticks from vegetation pools, including one *H. longicornis* and one *Dermacentor nuttalli*, were positive for viral RNA. We detected one pool positive for SFTSV RNA among the sheep raised at the patient’s home. SFTSV RNA was not detected in any of the collected ticks from hosts, pigs or cattle (Table 1).

### 3.4. SFTSV IgM and IgG Prevalence in Healthy Individuals

Table 2 shows the demographic characteristics of healthy individuals with SFTSV seropositivity. A total of 1169 serum samples were collected and tested for SFTSV IgM and IgG by indirect ELISA. From the 1169 individuals, 8 were positive for IgM and 20 were positive for IgG, giving seropositivity rates of 0.68% (8/1169) and 1.71% (20/1169), respectively. There were no statistically significant differences in IgM or IgG seropositivity rates between gender or among different age groups (Table 2). All SFTSV IgG seropositive samples were further tested for neutralizing antibodies by the microneutralization assay. Of the 20 individuals, 5 were positive for neutralizing antibodies, with seropositivity rates of 0.74% (5/1169). The titers of neutralizing antibodies were 1/32, 1/355, 1/672, 1/710 and 1/844, with a geometric mean titer (GMT) of 1/342. There were no statistically significant differences in neutralizing antibody rates between genders and occupations or among different age groups.

### 3.5. Molecular Characterization of SFTSV Strains

A total of 36 SFTSV-positive specimens with Ct values ≤ 28 were selected for next-generation sequencing. A total of 68 SFTSV genomic sequences were identified, comprising 21 L segments, 23 M segments and 24 S segments. Among them, the complete genome sequences of thirty-three patients and two ticks and the S segment of one sheep were successfully obtained. All sequences were submitted to the NCBI database and assigned GenBank accession numbers.

**Figure 4:**
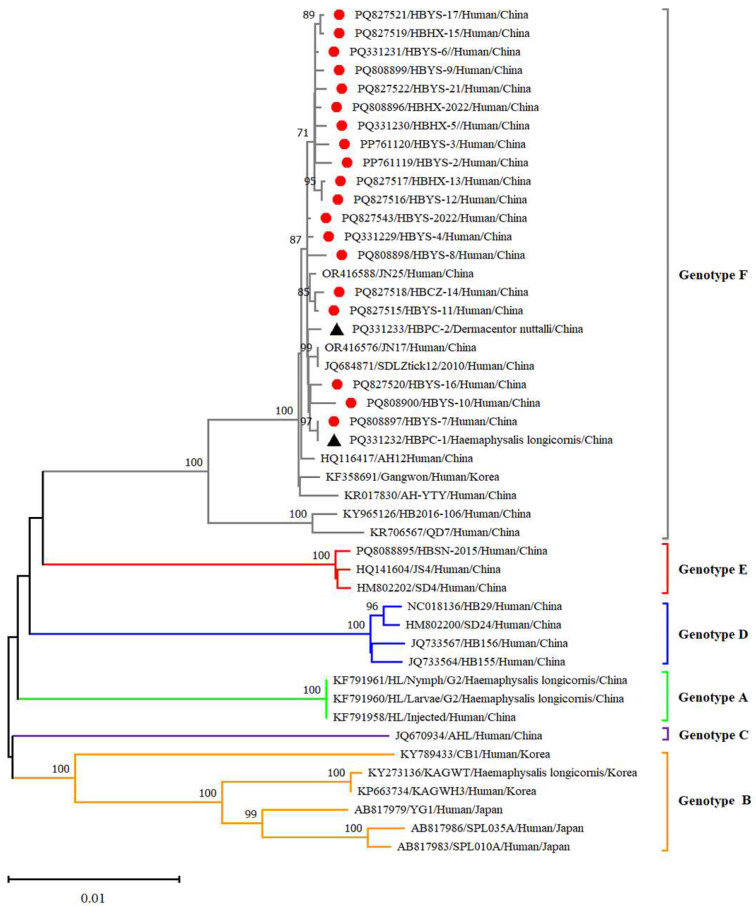
Phylogenetic analysis of SFTSV variants from patients and ticks. Maximum likelihood trees were constructed based on full-length of L segment. Red circles indicate the sequences of SFTSV variants obtained from patients with SFTS; black triangles indicate the sequences of SFTSV variants obtained from ticks; scale bar indicates nucleotide substitutions per site.

**Figure 5:**
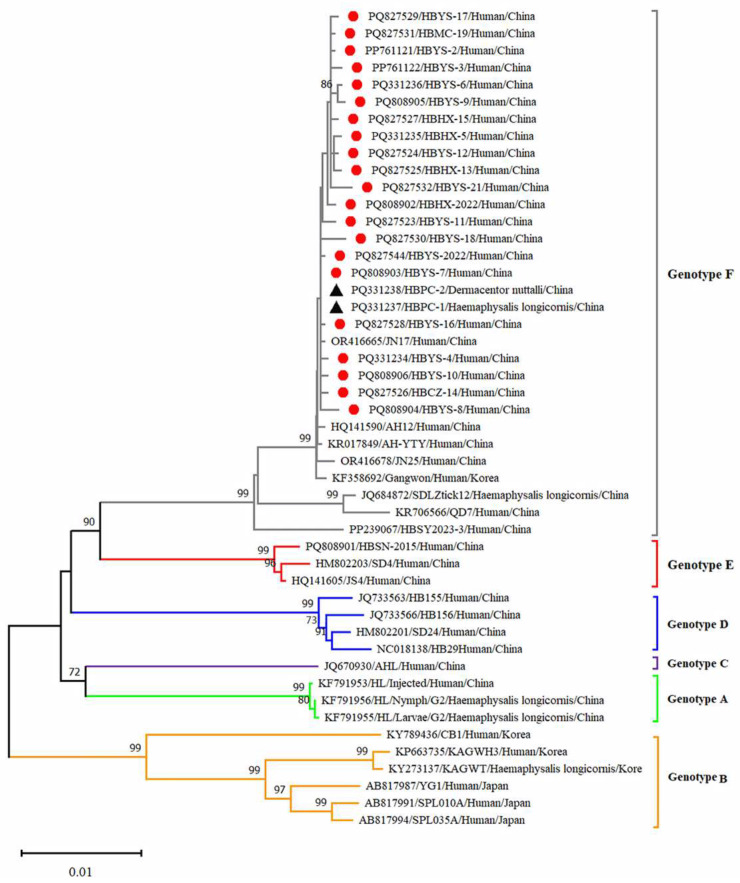
Phylogenetic analysis of SFTSV variants from patients and ticks. Maximum likelihood trees were constructed based on full-length of M segment. Red circles indicate the sequences of SFTSV variants obtained from patients with SFTS; black triangles indicate the sequences of SFTSV variants obtained from ticks; scale bar indicates nucleotide substitutions per site.

**Figure 6:**
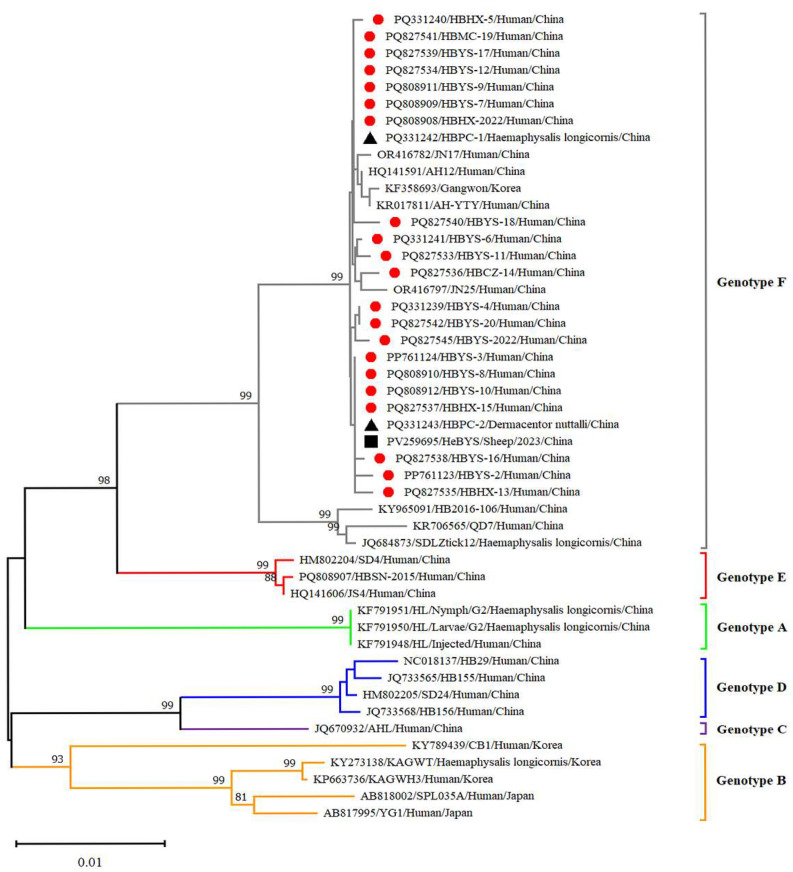
Phylogenetic analysis of SFTSV variants from patients and ticks. Maximum likelihood trees were constructed based on full-length of S segment. Red circles indicate the sequences of SFTSV variants obtained from patients with SFTS; black triangles indicate the sequences of SFTSV variants obtained from ticks; black square indicate the sequences of SFTSV strains obtained from sheep in Hebei Province, China. Scale bar indicates nucleotide substitutions per site.

Twenty-five reference strains representing different subtypes were downloaded from the NCBI database. These sequences, along with the SFTSV sequences obtained in this study, were subjected to comprehensive phylogenetic analysis. Phylogenetic analysis revealed that the L, M and S fragments of the SFTSV variants from patients, ticks and sheep were all clustered in the genotype F clade and closely related to variants from Shandong (JN17, JN25), Anhui (AH12/China/2010), AH-YTY/China/05/2012) and Republic of Korea (Gangwon/Korea/2012) (Figure 4, Figure 5 and Figure 6). The homology analysis revealed that the nucleotide and amino acid sequences derived from the tick specimens exhibited the highest sequence identity (99.9–100%) with variants from Shandong (JN17) compared with other reference variants (Supplementary Tables S1–S3).

## 4. Discussion

Prior to 2022, Hebei Province had only reported imported cases of SFTS, but locally acquired cases were first identified in 2022, with an upward trend in incidence observed thereafter. To confirm the existence of an SFTS endemic focus in Hebei Province, we conducted a multidisciplinary investigation integrating epidemiological surveillance, molecular characterization of SFTSV and seroprevalence analysis. Our findings conclusively demonstrate that Hebei Province has evolved into a newly established endemic focus for SFTSV transmission.

In China, SFTS cases have been predominantly reported in mountainous and hilly rural regions of seven high-incidence provinces [26,30]. However, our study revealed a striking epidemiological divergence. In total, 69 out of 75 locally acquired SFTS cases (92.0%) were clustered in Cangzhou City, a coastal alluvial–lacustrine plain in southeastern Hebei Province. Cangzhou City is characterized by the absence of traditional topographic risk factors and has no mountains or hills in the city. Yanshan and Haixing Counties, with the largest number of cases of Cangzhou city, are geographically adjacent to Dezhou and Binzhou cities of Shandong Province. As one of the high-incidence provinces for SFTS, Shandong Province has exhibited northward spatial diffusion of its epidemic focus [31]. Some scholars believe that the migration of ticks and the small animals that carry SFTSV may facilitate SFTSV viral range expansion, potentially increasing the risk of human infections in previously unaffected regions [32,33]. It can be inferred that SFTSV carried by ticks or host animals may disperse into the southern region of Cangzhou.

SFTSV has a certain latent infection rate in healthy individuals [34]. The IgM antibody detection rate of 0.68% and IgG antibody detection rate of 1.71% indicated the potential occurrence of subclinical or mild infections in Cangzhou. These findings highlight a latent risk of undetected transmission, which may contribute to the sustained viral circulation within the population. Results showed that 0.74% of serum samples were positive for SFTSV neutralizing antibodies, which was lower than the 1.71% of the IgG antibody positivity rate. This discrepancy may be attributed to a variety of antibodies, including neutralizing antibodies, being produced in viral infections, but the persistence durations of these antibodies in vivo vary. As the neutralization assay is recognized as the gold standard for detecting virus antibodies, we believe that the positive rate of 0.74% represented the lowest positive rate of SFTSV infection in the studied population. Individuals with positive neutralizing antibodies were predominantly observed in those aged over 40 years, indicating that middle-aged and elderly populations have an elevated risk of SFTSV infection. The level of latent SFTSV infection rate is closely related to the level of SFTS incidence [35], and ongoing dynamic serology surveillance is necessary to understand the dynamic pattern of SFTSV infection. Although no significant differences were observed among age groups, individuals aged ≥60 years exhibited a slightly higher IgG seroprevalence than those in other age groups, indicating that older adults have had more time to encounter the pathogen.

SFTSV was detected in patients, ticks and domestic animals in Cangzhou. The detection rate in patients was 61.73%, while that in free-living ticks and sheep was 1.98% and 4.54%, respectively. These findings are the first to demonstrate molecular evidence of SFTSV in ticks and domestic animals. Phylogenetic analysis of SFTSV sequences from patients, ticks and sheep (Ct ≤ 28) revealed that all sequences, including L, M, S segments clustered within the F subtype, were closely related to strains from Shandong (JN17,JN25), Anhui (AH12/China/2010), Zhejiang (AH-YTY/China/05/2012) and Republic of Korea (Gangwon/Korea/2012). It indicated that there was a close evolutionary relationship among those viruses isolated from patients with SFTS, ticks and domesticated animals. This close relationship demonstrated that the possible circulation of SFTSV has been established among livestock and ticks. The ticks and sheep, acting as transmission vectors and natural hosts, respectively, might play an important role in the transmission of SFTSV in Cangzhou. Additionally, homology analysis revealed that the SFTSV from ticks exhibited the highest sequence identity with JN17 variants from Shandong. Given the geographical proximity of the two regions, this finding implies a potential transmission route of SFTSV from Shandong Province to Cangzhou City. Further investigation is warranted to validate the possibility of virus introduction from Shandong. In light of the suitable habitats for ticks and the presence of animal hosts such as goats, cattle and pigs, the natural focus of SFTSV may have been established in Cangzhou City and is expanding geographically.

The highlight of our study is that the natural focus of SFTSV was not limited to hilly or mountainous areas. Epidemiological characteristics of SFTS in Hebei Province showed that SFTSV had naturally flourished in Cangzhou City, characterized by alluvial plains. The molecular evidence suggests a possible circulation of SFTSV among tick vectors and animal hosts, indicating the potential natural focus of SFTSV in plain areas.

## 5. Conclusions

In conclusion, the evidence of SFTSV circulation was detected among the ticks and host animals in Cangzhou City. Our findings highlight that the natural focus of SFTSV extends beyond hilly or mountainous areas, thus indicating a possible establishment of SFTSV circulation in the alluvial plain areas. In future research, intensive investigations should be conducted on the ecological conditions that underlie the maintenance of SFTSV transmission among animal hosts and ticks, which could provide a scientific basis for the prevention and control of SFTS.

## Figures and Tables

**Table 1:** Prevalence rates of SFTSV viral RNA in domesticated animals and ticks.

Species	Number of Ticks/Animals	Number of Tick Pools/Animals Sera	Number (MIR/%) of SFTSV-Positive Samples
Ticks from vegetation			
*Haemaphysalis longicornis*	95	7	1 (1.05%)
*Dermacentor nuttalli*	6	1	1 (16.67%)
Total	101	8	2 (1.98%)
Ticks from hosts			
*Haemaphysalis longicornis*	249	18	0 (0.00%)
*Rhipicephalus sanguineus*	52	5	0 (0.00%)
*Argas (Carios) sinensis*	1	1	0 (0.00%)
Total	302	24	0 (0.00%)
Domestic animals			
Pigs	31	31	0 (0.00%)
Cattle	20	20	0 (0.00%)
Sheep	22	22	1 (4.54%)
Total	73	73	1 (1.37%)

Note: MIR—SFTS virus minimum infection rate per 100 ticks (number of positive pools/total number of ticks × 100).

**Table 2:** Demographic characteristics of healthy individuals with SFTSV seropositivity.

Groups	No. Samples	Positive for IgM (%)	Positive for IgG (%)	Positive for Neutralizing Antibody (%)
Gender				
Male	544	4 (0.74%)	7 (0.60%)	2 (0.17%)
Female	625	4 (0.64%)	13 (1.11%)	3 (0.26%)
Total	1169	8 (0.68%)	20 (1.71%)	5 (0.43%)
χ^2^		1.00 *	1.09	1.00 *
*p*		0.56	0.29	0.56
Age (years old)				
0–19	192	1 (0.52%)	0 (0.00%)	0 (0.00%)
20–39	171	0 (0.00%)	3 (1.75%)	0 (0.00%)
40–59	398	5 (1.26%)	7 (1.76%)	2 (0.50%)
≥60	408	2 (0.49%)	10 (2.45%)	3 (0.74%)
Total	1169	8 (0.68%)	20 (1.71%)	5 (0.43%)
χ^2^		3.40	4.68	2.52
*p*		0.33	0.19	0.28
Occupation				
Farmer	976	7 (0.72%)	19 (1.95%)	5 (0.51%)
Others	193	1 (0.52%)	1 (0.52%)	0 (0.00%)
Total	1169	8 (0.68%)	20 (1.71%)	5 (0.43%)
χ^2^		1.00 *	0.23	1.00 *
*p*		0.61	0.13	0.41

* Comparison of groups was performed using Fisher’s exact test.

## Data Availability

All the sequences have been submitted to the Genbank.

## Supplementary Materials

The following supporting information can be downloaded at: https://www.mdpi.com/article/10.3390/v17060854/s1, Table S1: Homology analysis of L segment compared with reference strain of F genotype; Table S2: Homology analysis of M segment compared with reference strain of F genotype; Table S3: Homology analysis of S segment compared with reference strain of F genotype.
